# Understanding of professionalism among medical students in the first year of the COVID-19 pandemic – a qualitative monocentric study

**DOI:** 10.3205/zma001605

**Published:** 2023-04-17

**Authors:** Amelie Prade, Oliver Keis, Tim Sebastian, Wolfgang Öchsner

**Affiliations:** 1 University of Ulm, Medical Faculty, Ulm, Germany; 2 University Hospital Ulm, Department of Anesthesiology and Intensive Care, Ulm, Germany

**Keywords:** professionalism, professional identity formation (PIF), COVID-19 pandemic, medical students

## Abstract

**Objective:**

The existing literature indicates that medical students’ understanding of professionalism is influenced by internal and external factors. Therefore, this study aimed to evaluate whether the early phase of the pandemic affected the understanding of professionalism among medical students at the University of Ulm.

**Methods:**

In May and June 2020, semi-structured telephone interviews were conducted with 21 students (in the 8^th^ and 9^th^ semester) at the Medical Faculty of the University of Ulm. The interviews were transcribed and analyzed by a qualitative content analysis according to Mayring.

**Results:**

The results showed shifts in students’ perception of the importance of certain aspects of medical professionalism. Not only competency in the disciplines hygiene, virology, and microbiology came to the fore, but also personal qualities such as “radiating a sense of calm”, empathy, and altruism; communicative competency; and the capacity for reflection. The students also perceived changes in the expectations placed on them. More emphasis was placed on their roles as scientific or medical advisors and as helpers in the health care system, a change that was sometimes emotionally stressful. With respect to the study objective, both limiting and supporting factors were named. For example, the clarification of the relevance of the medical professional was motivating.

**Conclusion:**

The study showed that students’ understanding of professionalism depends on context, as was suggested by earlier studies in experts. The perception of changed role expectations may thereby also play a role. One consequence of the findings may be to address such dynamics in suitable curricular events and discuss them with students to prevent them proceeding in an uncontrolled manner.

## Zusammenfassung

**Zielsetzung:**

Gemäß der vorhandenen Literatur muss davon ausgegangen werden, dass das Professionalitätsverständnis von Medizinstudierenden internen wie externen Einflüssen unterliegt.

Ziel der Studie war deshalb, das Professionalitätsverständnis Ulmer Medizinstudierender unter dem Einfluss der beginnenden Pandemie zu untersuchen.

**Methoden:**

Im Mai und Juni 2020 wurden 21 semi-strukturierte Telefoninterviews mit Studierenden (8./9. Semester) der Medizinischen Fakultät Ulm durchgeführt. Die Interviews wurden transkribiert und mittels qualitativer Inhaltsanalyse nach Mayring ausgewertet.

**Ergebnisse:**

Es zeigten sich bei den Studierenden Verschiebungen in der Bedeutungswahrnehmung einzelner Aspekte von ärztlicher Professionalität. Kompetenzen aus den Fächern Hygiene, Virologie und Mikrobiologie traten in den Vordergrund, aber auch persönliche Qualitäten wie „Ruhe ausstrahlen“, Empathie, Altruismus, sowie kommunikative Kompetenzen und Reflektionsfähigkeit. Auch wurden potentielle Erwartungshaltungen an die Studierenden als verändert wahrgenommen. Rollenzuschreibungen als wissenschaftliche bzw. medizinische Ratgeber, sowie als Helfende im Gesundheitswesen traten in den Vordergrund, zum Teil war dies emotional belastend.

In Bezug auf die Studienmotivation wurden sowohl hemmende als auch fördernde Faktoren benannt. Motivationsfördernd war beispielsweise die Verdeutlichung der Relevanz des ärztlichen Berufs.

**Schlussfolgerung:**

Die in früheren Studien auf Expert*innenniveau postulierte Kontextabhängigkeit des Professionalitätsverständnisses bestätigt sich auch auf der Ebene der Studierenden. Dabei könnte auch die Wahrnehmung veränderter Rollenerwartungen eine Rolle spielen. Um derartige Dynamiken nicht unkontrolliert ablaufen zu lassen, könnte eine Konsequenz darin bestehen, diese in geeigneten curricularen Veranstaltungen zu thematisieren und mit den Studierenden zu diskutieren.

## 1. Introduction and objective

The terms professionalism and development of professionalism can be defined with different concepts [1], [2]. Their multidimensionality can probably be most closely approximated with the concept of professional identify formation (PIF).

According to van de Camp et al., three different domains are involved:

1. the intrapersonal domain, which includes one’s own perception and the emotional stress of a situation, for example;
2. the interpersonal domain, which includes expectations concerning roles, for example; and
3. the publicly accessible domain, which refers to the position within society, for example [1], [2], [3].

If, as can be assumed, being confronted by a novel global event such as the COVID-19 pandemic influences one or more of these domains, the pandemic situation could be assumed to have effects also on medical students’ understanding of professionalism.

In the past two years, various research groups have studied different aspects of the pandemic [4], [5], [6], [7], [8], [9], but nevertheless, many questions remain in educational research on this topic. The aim of this study was to gain insights into students’ understanding of various aspects of medical professionalism during the early phase of the COVID-19 pandemic.

## 2. Situation and methods

### 2.1. Situation at the time of the survey 

The first cases of COVID-19 infection in Germany were reported at the end of January 2020 [10]. The national state of emergency was declared at the end of March 2020 after the WHO had declared the pandemic [https://covid19.who.int/]. In the study period from May to June 2020, Germany was experiencing the first wave, with a peak level of 170,000 infected people [https://covid19.who.int/], [11], [12]. At that time, neither sufficient protective equipment for medical staff nor enough masks for the population and neither curative treatments nor vaccines were available. 

At first, in medical degree courses, as in other degree courses, practical ways to continue the degree program were being sought for, and a lot of things were still being improvised [13]. At the survey site, the summer semester 2020 could only start later than planned, and some internships and exams were delayed or canceled. Social contacts with other students and lecturers were lacking because of the transition to digital teaching formats and restrictions on gatherings in professional and private settings. 

Medical students were publicly called on to become involved in the health care system to help cope with the pandemic [10], [13]. As a result, they took on a kind of special role in patient care and at testing centers and public health departments for example [14].

#### 2.2. Sample composition and selection

This qualitative study was performed from May to June 2020 at the Medical Faculty of the University of Ulm.

The survey target group comprised medical students at the University of Ulm in the 8^th^ or 9^th^ semester. At the time of the survey, these students had both experience as medical students and initial experiences in patient care, so they were assumed to have at least a tacit understanding of professionalism.

After approval of the project by the ethics committee of the University of Ulm, all 392 medical students in the 8^th^ or 9^th^ semester at the university were informed by email about the study and invited to participate voluntarily. Exclusion criteria for participation were medical training before the current medical degree course, being sick with COVID-19, and COVID-19 related severe illness or death of close friends or family.

A total of 34 students agreed to participate, but 4 of them fulfilled exclusion criteria. From among the remaining 30 students, 14 women and 7 men were randomly chosen to participate so that the sex distribution was representative of that in the medical degree course at the University of Ulm. The chosen 21 students provided written consent for the processing of their data.

#### 2.3. Data collection and analysis methods

For the semi-structured interview, a guideline interview was prepared with the help of the SPSS method according to Helfferich [15]. When selecting the questions for the interview, we used the above-mentioned domains, among other things, as guidance and always considered the relevance of the domains for the understanding of professionalism (see attachment 1 ). This approach yielded 17 open questions, each with 2 to 3 refining questions. The interviews were conducted by telephone by two trained interviewers and lasted a mean of 28 minutes. The conversations were recorded electronically and subsequently transcribed with the software F4 Transcript. Then, the software MAXQDA 2020 was used to code the statements by performing a qualitative content analysis according to Phillip Mayring [16]. First, the individual statements were clustered into subcodes and then into codes, which revealed the respective core statements in the overall context of the interview. The prevalences of the individual codes and the numbers of students that contributed to the respective codes are depicted in the tables in the Results section.

## 3. Results

### 3.1. Understanding of medical professionalism during the early phase of the pandemic

Most of the participants stated that their understanding of medical professionalism had not been fundamentally changed by confronting the pandemic. However, they perceived that the weighting of certain aspects of professionalism increased (Quote 1). Besides professional competency in the fields of hygiene, virology, and microbiology, this change was noticed for personal qualities such as “radiating a sense of calm” and communicative competencies (see table 1 (Tab. 1)). For about half the participants (10/21), the consequence of this development was that they would put more effort to learn these competencies in the future.

*„(…) the aspects, which have already been named, are always important – but are of course now really emerging (…)“ – (Quote 1)*

#### 3.2. Students’ perception of the pandemic situation

Most of the participants (18/21) stated that they were able to more realistically understand and evaluate the pandemic situation than the lay population and that they assumed that all medical students would have this specific understanding. This different understanding was explained by both their acquired expert knowledge (in particular in the fields of hygiene, microbiology, virology, and epidemiology) and the experiences gained through their pandemic-related assistance in the health care system.

The question about possible consequences of this specific understanding was answered in two divergent response categories. In the majority of participants (10/18), the physician-specific understanding of the situation resulted in easier acceptance of the Corona-related limitations and rules of conduct (Quote 2). In addition, 6 students reported that when they had observed careless behavior they had reacted by actively addressing the respective person. However, in a smaller number of students (4/18), their understanding of the situation resulted in them adhering less strictly to the rules mandated during the pandemic. These students stated that because of their special background knowledge, they decided themselves which rules and measures they accepted as meaningful for themselves (Quote 3).

*“you took it a bit more seriously. (…) completely convinced that that is the right way. And you should therefore stay at home as strictly as possible and (…) should meet few people.” (Quote 2)*

*„(…) I believe that therefore [because of the medical degree] I have not just stubbornly (…) followed every rule, but I have balanced the pros and cons of each rule separately (…)“ (Quote 3)*

#### 3.3. Subjective perception of expectations 

More than half the interviewed students stated that increased expectations were placed on medical students at the start of the pandemic. These expectations were mainly related to areas that stem from the cognitive aspects of medical training, such as scientific and medical knowledge and understanding. However, in addition they also perceived increased expectations with respect to social interactions, such as empathetic interactions with patients, as well as with people from their own environment (see table 2 (Tab. 2)). For about a third of the participants, these increased expectations were accompanied by an increased social esteem of medical students.

Some students also described the phenomenon that although the people around them placed no or hardly any pressure on them, they had built up an internal weight of expectations themselves (Quote 4).

*“So, in my case no one expected that I would help or work anywhere. But I expected it from myself.” (Quote 4)*

#### 3.4. Emotional stresses 

Only two students stated that the pandemic did not cause increased emotional stress.

The types of emotional stress described by the remaining students were mainly caused by the uncertainty of the situation and by feelings of helplessness (Quote 5), as well as by the measures that became necessary to curb the pandemic (see table 3 (Tab. 3)). In addition, the above-mentioned pressure placed on students by people around them was named as a stressful factor, in particular if the student’s own commitment was perceived as being insufficient.

When students were asked how they dealt with the pandemic-related stress, only a few concrete coping strategies were named. These were exercising (3 codes, 3 students), mindfulness or yoga exercises (3 codes, 1 student), and conversations (2 codes, 2 students).

*“So, personally I felt overwhelmed (…) mainly (…) with these people’s lack of prospects and this uncertainty that no one so to say knows what it will be like in half a year, (…) , so what can I do, what can I not do, what are the next steps here, what are the next steps there.” (Quote 5)*

#### 3.5. Effects of the pandemic on motivation for studying and career plans

About half of the study participants’ observations on the effect of the start of the pandemic on their own motivation for their degree were positive and about half were negative.

In particular, the uncertainty about the further course of their studies and the initially not yet fully developed content and technology of online teaching were named as factors that clearly decreased motivation.

As a factor that clearly increased motivation was the population’s increased perception at the start of the pandemic of the relevance of the medical profession, which led to the efforts of studying being experienced as being even more meaningful (Quote 6).

*“But also motivating. (…) this confirmation, you’re doing something meaningful here. Definitely, yes.” (Quote 6)*

The question whether there were direct effects on the own career plans, as far as intended specialty or intended type of employment was concerned, was predominantly negated.

## 4. Discussion

### 4.1. Understanding of medical professionalism in the context of the pandemic

On the one hand, the students we interviewed stated that their perception of medical professionalism had not fundamentally changed by the occurrence of the pandemic situation. This could initially suggest that medical professionalism can be understood as a kind of entrenched value system.

However, on the other hand, the interviewees stated that at the time of the survey, i.e., shortly after the start of the pandemic situation, intra- and interpersonal aspects such as “radiating a sense of calm”, “altruism”, “empathy”, “good explanations”, and “reflective communication” clearly came to the fore in the understanding of professionalism, as did the subjective weighting of specific professional competency in the fields of hygiene, virology, and microbiology. The students’ explicit willingness to consequently learn more about these fields indicates a concrete impact on their own learning behavior.

Thus, on the one hand fundamental changes were negated, but on the other hand shifts in priorities were mentioned. To place these results, which at first glance appear to be potentially contradictory, in a meaningful overall context, it is worthwhile taking another look at the systematic review by van de Camp et al. mentioned in the Introduction [1]. At that time, the authors of the review noticed that during the validation process, each of the experts involved in validating the three domains had added their own, completely different items within the domains. The fact that the experts involved in the valuation were working in different occupational situations led the authors to hypothesize that the understanding of professionalism may or even probably depends on the context. The context dependence of the understanding of medical professionalism assumed for the expert milieu is supported by the results of our study also for the student milieu. The finding that the students mentioned changes in the subjective weighting of individual aspects of professionalism only a few months after the start of the pandemic indicates that this context dependence has an effect relatively quickly, at least in the case of substantial external influences such as the outbreak of the pandemic. Such shifts in the understanding of professionalism among students may be facilitated by the fact that although students have an implicit understanding of medical professionalism [17], [18], this understanding is not yet firmly established. Hilton and Slotnick have termed the condition of a not yet fully developed understanding of professionalism as “proto-professionalism” [17].

#### 4.2. Subjective perception of expectations and role assignments

The theoretical framework of professional identity formation views the development of professional identity as a process on both an individual and a collective level, whereby the two levels mutually influence each other. Other influences stem from interactions with patients, peers, and role models; external events, such as the occurrence of a pandemic situation, as in the current case; and last but not least, also the perception of expectations from the social environment and society [2], [3], [18]. In the context of the pandemic situation, perceiving such expectations as being changed led to changes in the ascribed roles of medical students. In particular, special emphasis was placed on the advisory role with respect to medical-scientific background knowledge or advice in case of symptoms and on the helping role, e.g., while working in the health care system. The same was true for the role associated with empathetic behavior. The finding that changed priorities in students’ own understanding of their role are accompanied by changed priorities in their understanding of medical professionalism, as we saw in our results, provides further support for the above-mentioned hypothesis about context dependence.

#### 4.3. Emotional stresses 

In particular the uncertainties and behavioral rules associated with the pandemic situation were perceived as being emotionally stressful and were clearly not very specific and not very different from the widespread stresses in the general population. The above-mentioned, changed roles resulted in emotional stresses when the students perceived the associated expectations as not being fulfilled.

In contrast to other studies [19], the participants in our survey named hardly any concrete coping strategies to help them deal with such stresses better.

#### 4.4. Effects of the pandemic on motivation for studying and career plans 

A study performed in Geneva, Switzerland, reported mainly negative effects of the start of the pandemic on students’ level of motivation, whereas the increased need for students to assist in the health care system was perceived as a positive confirmation of the choice of degree and profession [20]. Similarly, the students we interviewed described contrasting effects on their motivation. The ad hoc organizational, technical, and to some extent content-related curricular changes led to uncertainties and loss of motivation among the students. In contrast, the sudden further increase in the standing of the medical profession and medical degree course among the population increased motivation. The latter effect is in line with the findings of a recent Swiss study on the level of motivation among Generation Z, where aspects of external motivation such as esteem and prestige were weighted slightly higher by future medical students than by students of other degree courses [21].

We found no effects of the pandemic on the students’ career plans, a finding that is similar to current study results of other research groups [22]. Although an American study showed an impact on career choice, this was the case mainly among younger students; such effects were not detectable among students who were further along in their degree [20].

#### 4.5. Limitations

The present study has the following limitations: It was a qualitative study at a single center that analyzed the subjective expression of opinions of a certain group of students. Although the sample size appears to be good for a qualitative study, the results cannot be generalized as statistically representative. The advantage of the qualitative approach is that it allowed the phenomenon of the COVID-19 pandemic, which was new at the time of the survey, and the associated changes in students’ understanding of professionalism to be approached openly. In our opinion, this enabled the study to reach a depth that would be difficult to imagine with a quantitative survey.

Because the survey was performed only at a single time point of the pandemic (cross-sectional design), no statements can be made about how the understanding of professionalism would have developed longitudinally over the various semesters. The question whether the global event of the COVID-19 pandemic had a long-lasting effect on the surveyed students can also not be answered.

Future studies need to evaluate whether the context dependence of the understanding of professionalism in the student phase of proto-professionalism may be higher than in later phases of the professional career. This question cannot be answered by our study, in which interviews were conducted at only one point in time. Within the medical degree, the study was limited to students in the 8^th^ or 9^th^ semester because they have sufficient experience of studying and also have their first experiences with patients.

## 5. Take-home messages and conclusion

The results of our study can be summarized as follows:

1. The outbreak of the pandemic did not fundamentally change the understanding of professionalism among the surveyed students. However, it was accompanied by changes in the value of certain aspects of professionalism.
2. The expectations of the role of medical students was perceived as having changed, in particular as far as the increased relevance of the advisory and helping role was concerned. These changes in the perception of expectations may have contributed to changes in the students’ understanding of professionalism because of context dependence.
3. The effects on student motivation were very different. Pandemic-related uncertainties and irregularities in the learning procedures had a negative effect on motivation. In contrast, the increased perception of the relevance of the medical profession had a positive effect on motivation.

The following may be conclusions with respect to curricular measures: 

Students‘ understanding of professionalism can be influenced by external events, presumably also by changes related to role expectations placed on the students. Such influences are not always predictable. To prevent such dynamics from proceeding in an uncontrolled manner, it appears to be meaningful to address them explicitly in curricular sessions on the topic of medical professionalism and to discuss possible consequences with students.

## Acknowledgement

Support was kindly provided by the Working Group of Teaching Research of the University of Ulm in the form of covering costs.

## Competing interests

The authors declare that they have no competing interests. 

## Supplementary Material

Guideline interview

## Figures and Tables

**Table 1:**
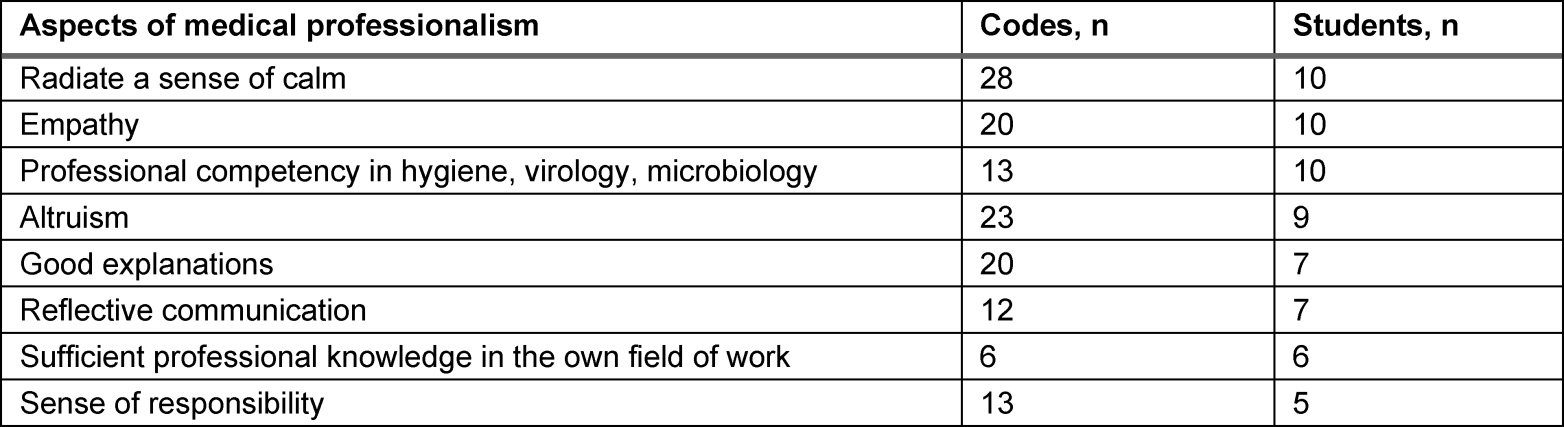
Aspects of medical professionalism perceived as being important

**Table 2:**
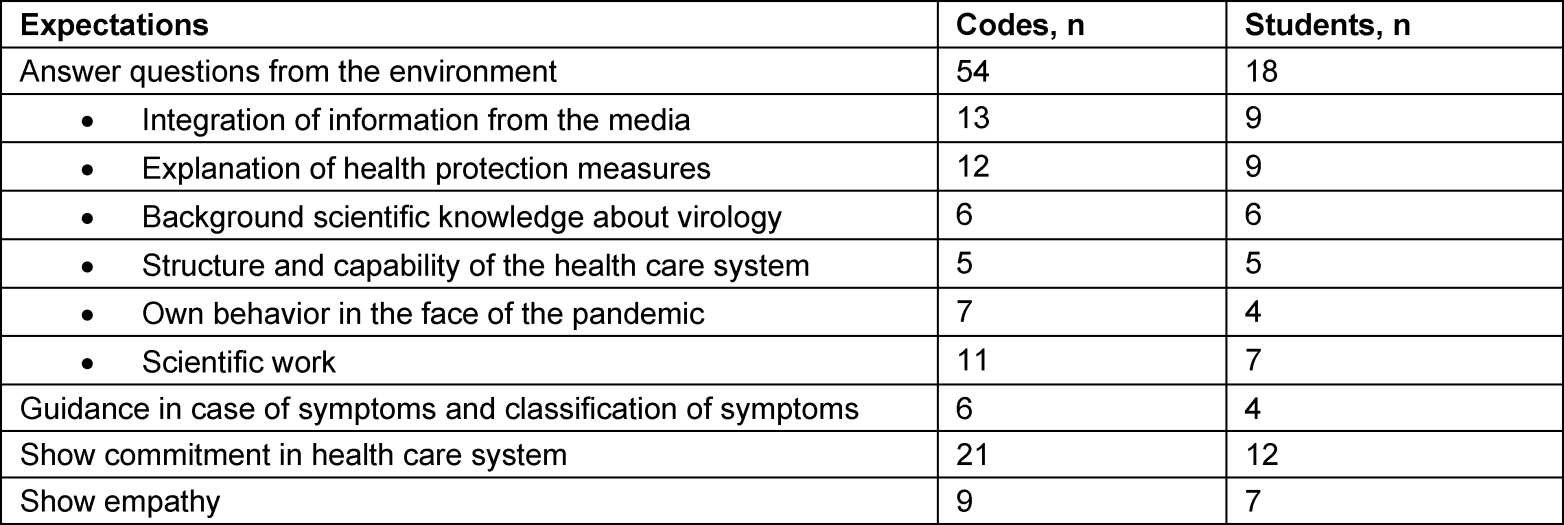
Expectations perceived by the medical students

**Table 3:**
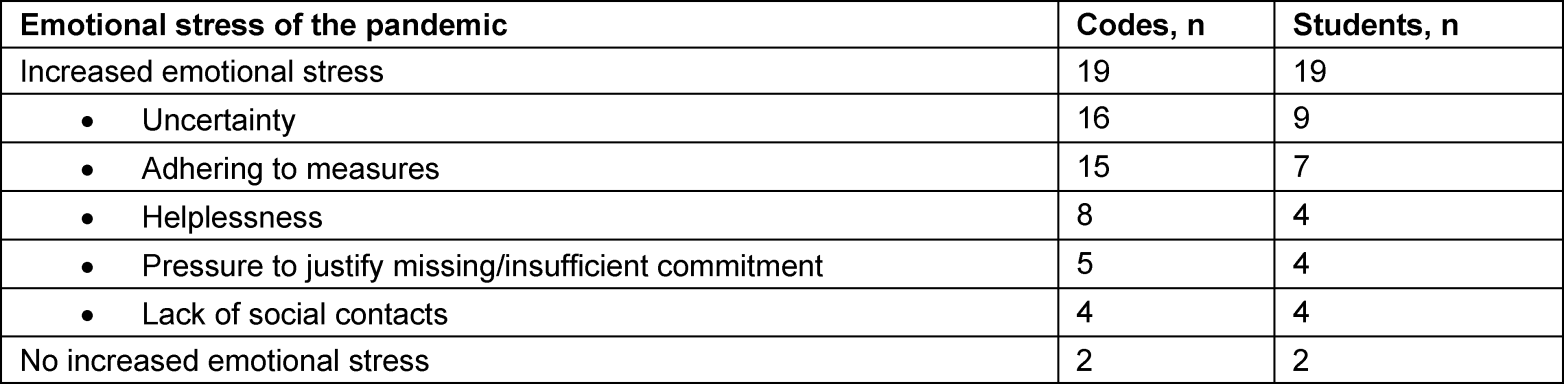
Causes of emotional stress
