# Supplementary material for: Understanding of professionalism among medical students in the first year of the COVID-19 pandemic – a qualitative monocentric study
Source: GMS J Med Educ. 2023 Apr 17;40(2):Doc23. doi: 10.3205/zma001605 (PMC10285364; doi:10.3205/zma001605)
Supplement: Guideline interview [file JME-40-23-s-001.pdf]

Attachment 1: Guideline interview

Demographic, person-related data:

- Greeting and information about the procedures
- Informed consent: Agrees with recording of conversation on tape, subsequent transcription, and anonymized analysis of the data?
- NAME OF PREVIOUS SEMESTER and BLOCK/COURSE (state before the start of the interview)

<u>First step: Personal impressions</u>

Aims of questions: ice-breaking phase, warm-up phase, familiarization with the topic, linking of the topic with personal experience

Important point: "Bad" personal experiences could be activated (e.g. death in the family)—if that happens, it must be dealt with carefully by the moderator!

- How have you <u>experienced (and understood)</u> the pandemic (personally)? How did you feel about it?
  - (Which aspects) What has (of the pandemic have) thereby <u>made the biggest impression</u> on you?

Perception of the pandemic by medical students, perhaps in contrast to students in other degree courses

Aims of questions: Triggered effects are closely related to the respective understanding, and in turn, understanding is influenced by prior knowledge and experience.

- Do you have the impression/feeling that the Corona pandemic was or is <u>understood differently by medical students</u> than by students of other degree courses?
  - If yes, in your opinion what are the differences between the respective understanding of medical students and that of other students? How did you reach this assessment?
  - Did the possibly different understanding also result in different actions or different behaviors? Can you name concrete examples?

Type and concrete effects of medicine-specific competencies

Aims of questions: Capture subjective value of "physician-specific" competencies and effects of competencies on the person and their environment

- Which medical knowledge <u>or which competencies were especially useful or valuable for you personally during the pandemic</u> (for yourself or for other people)?
  - Which competencies that you learned during your studies have helped you most during the pandemic so far and in what way (for yourself or for other people)?

  - Did you have the feeling that as a medical student you can/could interpret the information provided by the media better than many other people?

- Were there situations in which you actively contributed your medical knowledge or competency to your environment on your own initiative?
  - How exactly did these situations unfold? You are welcome to give examples!
  - Were you able to use your knowledge, for example, to inform or advise other people?

Role expectations and categories of professionalism in the context of the pandemic
Aims of questions: This part asks about some essential components of "professionalism in the context of the pandemic": roles, role characteristics ("qualities"), role modeling ("role models"), dealing with unknown situations, dealing with feeling overwhelmed

- Do you have the feeling that your environment has or had special expectations—more than is otherwise the case—of you as a medical student?
  - What were those expectations?
  - Which of the competencies assumed by the environment were then mainly asked about or made use of?
  - To what extent could you actually fulfil these expectations?

- Are there other aspects in which during the pandemic your role or your function as a medical student has perhaps changed somewhat—be it in your own perception or in the perception of your environment?

- In your opinion, which qualities of a physician were particularly important in the overall course of the pandemic so far?
  - If necessary, to clarify/check: "Qualities" refers also to competencies, as well as to knowledge or characteristics! Perhaps inquire specifically if only one or two of these categories are named spontaneously.
  - Perhaps dig deeper: Qualities at the start of the crisis PLUS qualities at the current time—may be different.

- If you yourself needed additional information or some good advice, what did you do, how did you obtain advice and information?
  - Which people and which media (be sure to ask about both!!) were your most important advisors and sources of information?
  - Why were these in particular so important?
  - Why did you especially trust these people or sources?

- Do or did you have personal role models for professional behavior in a crisis?
  - Which ones were they specifically in which situations?

- Were there situations associated with the pandemic in which you simply felt overwhelmed?
    - Which were they specifically?
    - How did you react to them, how did you deal with them?

Impulses for change (suggested or specifically initiated by the pandemic)
Aims of questions: Recording impulses for change (related to studying, occupation, or oneself)

- Has your previous understanding of "(medical) professionalism" changed through the current experiences, was this understanding modified?
    - Has your understanding of what makes a "good physician" changed through the current experiences?
    - If yes, how would you describe the influences or changes?

- Are there competencies that you personally underestimated in your studies so far and for which you now particularly appreciate the importance and relevance—if yes, which?
    - Would you yourself be prepared to put considerably more effort into learning about the named competencies (e.g., to invest more studying time, spend an internship there, etc., etc.)?

- Does the pandemic have a noticeable effect on your own motivation for learning? (Be it rather strengthening, be it rather limiting or even demotivating)
    - Have you noticed these kinds of effects among your fellow students? How are such effects expressed, for example?

- Has the pandemic affected your own career plans? (no examples)
    - For example, regarding the choice of specialty?
    - For example, regarding the choice between hospital or office-based work?

- Every new situation is accompanied by surprising or learning effects. Which surprising or learning effects have you noticed about yourself during the pandemic?

- In summary: If you had to write down as keywords the most important pandemic-related stimuli, impulses, or changes to your career path as a physician, what would those keywords be? In conclusion, describe as keywords the most important pandemic-related impulses, stimulations or changes to your career path as a physician!

- Is there anything else that you would like to say about the topics of our interview that has not yet been mentioned— additions, suggestions, afterthoughts?

Say thank you and goodbye and briefly discuss the further procedures (sending of forms, returning completed forms, then transfer of reimbursement)
